# Identification of ZG16B as a prognostic biomarker in breast cancer

**DOI:** 10.1515/med-2021-0004

**Published:** 2020-11-25

**Authors:** Haotian Lu, Chunying Shi, Xinyu Liu, Chen Liang, Chaochao Yang, Xueqi Wan, Ling Li, Ying Liu

**Affiliations:** School of Basic Medicine, College of Medicine, Qingdao University, Qingdao, 266071, China; Department of Human Anatomy, Histology and Embryology, School of Basic Medicine, College of Medicine, Qingdao University, Qingdao, 266071, China; Institute for Translational Medicine, The Affiliated Hospital of Qingdao University, College of Medicine, Qingdao University, Qingdao, 266071, China

**Keywords:** ZG16B, breast cancer, database, biomarker, prognosis

## Abstract

Zymogen granule protein 16B (ZG16B) has been identified in various cancers, while so far the association between ZG16B and breast cancer hasn’t been explored. Our aim is to confirm whether it can serve as a prognostic biomarker in breast cancer. In this study, Oncomine, Cancer Cell Line Encyclopedia (CCLE), Ualcan, and STRING database analyses were conducted to detect the expression level of ZG16B in breast cancer with different types. Kaplan–Meier plotter was used to analyze the prognosis of patients with high or low expression of ZG16B. We found that ZG16B was significantly upregulated in breast cancer. Moreover, ZG16B was closely associated with foregone biomarkers and crucial factors in breast cancer. In the survival analysis, high expression of ZG16B represents a favorable prognosis in patients. Our work demonstrates the latent capacity of ZG16B to be a biomarker for prognosis of breast cancer.

## 1 Introduction

Breast cancer is the most commonly diagnosed cancer in women, which is one major cause of cancer death especially in young women, second only to lung cancer [1,2,3,4]. Based on the expression of estrogen receptor (ER), progesterone receptor (PR), and human epidermal growth factor receptor 2 (HER2), breast cancer is divided into Luminal A, Luminal B, Basal-like, and HER2-positive subtypes [5]. Many therapies have been developed and used to detect and treat breast cancer [6,7,8]. However, due to the complex interactions between the environment and hereditary factors, it’s still difficult to diagnose or prevent breast cancer at initial stage [9]. It has been reported that the abnormal increase of biomarkers in tumorigenesis can be detected in blood, urine, and tissue and then help predict tumor’s grade malignancy, behaviors, and prognosis [10]. In previous studies, multiple kinds of conventional biomarkers related to early diagnosis and prognosis for breast cancer have been developed, such as uPA [11], Rs/DJ-1 [12], and PAI-1 [13]. And recently, circulating miRNAs [14], serum uPAR [15], KiSS1 [16], CD24 [17] etc. also have been recognized as strongly associated with breast cancer development. In order to improve the early detection, diagnosis, and prognosis or even discover therapeutic targets of breast cancer, more specific biomarkers need to be identified.

Zymogen granule protein 16A (ZG16A), also known as ZG16 or ZG16p, is a soluble lectin expressed in pancreatic acinar cells and digestive tract, which mediates the condensation of pancreatic enzymes to the zymogen granule membrane [18,19]. Zymogen granule protein 16B(ZG16B), also identified as Pancreatic adenocarcinoma upregulated factor, is a paralog of ZG16A which has a 65.5% of similarity and 36% identity, first found to be overexpressed in pancreatic ductal adenocarcinoma [19,20,21]. Both of these two zymogen granule proteins exist in human alimentary system and have the same structures such as β-prism fold and glycosaminoglycan-binding site, suggesting their potential functional similarity [19,21].

ZG16A has been recognized as a mucus ingredient in colon fluid which blocks bacteria and upregulates in colorectal cancer as a biomarker [22,23]. ZG16B was first discovered to act as a growth factor overexpressed in pancreatic cancer, which enhances tumor proliferation and helps escape from innate immune system by intriguing TLR-mediated ERK signaling, inhibiting TLR-mediated NF-kappa B signaling and keeping β-catenin stable through phosphorylation [21,24,25]. Furthermore, ZG16B enhances angiogenesis and vascular permeability and then promotes tumor progression and metastasis of pancreatic cancer by stimulating CXCR4 expression and FAK activation [26,27,28]. Additionally, ZG16B helps pancreatic cancer cells to resist oncolytic parvovirus H-1 infection via IFNAR-mediated signaling [29]. As a special factor in pancreatic cancer, ZG16B promotes activation and maturation of DCs through TLR4 signaling pathway to mediate immune system activation; meanwhile, it could also increase and activate MDSCs to benefit tumor formation, showing a double-sided effect on the immunotherapy [30,31]. And in chemotherapy, ZG16B enhances the effect of gemcitabine and 5-FU in pancreatic cancer [32,33].

In addition, ZG16B has been identified as a biomarker highly expressed in colorectal cancer and enhancing the migration and invasion, leading to a poor prognosis [20,34,35,36]. ZG16B is also confirmed to exist in HeLa cells and upregulates in cervical cancer [37,38]. Moreover, ZG16B can also have effect and be a biomarker for early diagnosis and prognosis of prostate cancer [39], oral squamous cell carcinoma [40], and especially ovarian cancer [41,42,43]. Besides, ZG16B is correlated with the prognosis of atherosclerosis and acute coronary syndrome [44], and it is demonstrated to be abundant in the reflex tears as the key point in the ocular surface protection, maintaining the tear film stability [45,46]. ZG16B has been detected as a biomarker in various malignant tumors; however, the association between ZG16B and breast cancer has not been noticed yet.

In this report, we found that ZG16B expressed highly in breast cancer, and depth analysis was conducted further to clarify the possible effect of ZG16B in breast cancer through public medical databases. Expression levels in different conditions, possible mechanisms of action and the effect of prognosis of ZG16B in breast cancer were presented, demonstrating its potential value to be a biomarker for breast cancer in clinical practice.

## 2 Materials and methods

### 2.1 Ethics statement

Our work has been approved by the Ethics Committee and Institutional Review Board of Qingdao University, China. Informed consent for publication was not required, as all patient data used in the study were obtained from publicly available databases.

### 2.2 Oncomine database analysis

Oncomine database (https://www.oncomine.org), which provides 715 datasets and 86,733 samples, was referred to analyze the expression pattern of ZG16B mRNA in different types of cancers. Pooled meta-analysis in different subtypes of breast cancer and gene co-expression analysis of ZG16B were conducted by Oncomine. The threshold of *p*-value was fixed to 1 × 10^−4^. The threshold of fold change was fixed to 2.

### 2.3 CCLE analysis

The expression level of ZG16B in different cell lines was analyzed by Cancer Cell Line Encyclopedia (CCLE, https://portals.broadinstitute.org/ccle), a database offering the expression level sorting of 84,434 genes in 1,457 cancer cell lines.

### 2.4 Ualcan analysis

Ualcan (http://ualcan.path.uab.edu/analysis.html) is a user-friendly cancer database based on TCGA database. Data from TCGA database were obtained through Ualcan to analyze the expression discrepancy of ZG16B in diverse molecular subtypes of breast cancer, as well as gender, age, cancer stages, and node metastasis status. The promoter methylation status of ZG16B was also analyzed using Ualcan.

### 2.5 STRING analysis

STRING (https://string-db.org) is a database that collects known and predicted protein–protein physical and functional interaction information, the data of which originate from computer prediction, knowledge sharing between organizations, and other databases. A protein network was drawn by STRING to find the interaction between ZG16B and other proteins.

### 2.6 Survival analysis

The Kaplan–Meier plotter (http://kmplot.com/analysis) is an online graph plotter including the survival data of patients with multiple types of cancers to draw survival curves. The effect of ZG16B on prognosis in breast cancer was analyzed by Kaplan–Meier plotter on the scale of relapse-free survival (RFS). ZG16B Affy ID: 228058_at. The Probe set option is the user selected probe set. The cut-off value is determined by the median.

### 2.7 Statistical analysis

Students *t*-tests were performed to detect the statistical difference of ZG16B mRNA expression between breast cancer and normal tissues, as well as the mRNA expression level and promotor methylation level in different clinical indicators. Log-rank test and hazard ratio (HR) analyses were conducted to examine the statistical difference of survival curves in different subtypes of breast cancer with low or high expression of ZG16B. Data were presented as the mean ± standard error of mean, and *P* < 0.05 was considered to be statistically significant.

## 3 Results

### 3.1 ZG16B expression in breast cancer

**Figure 1:**
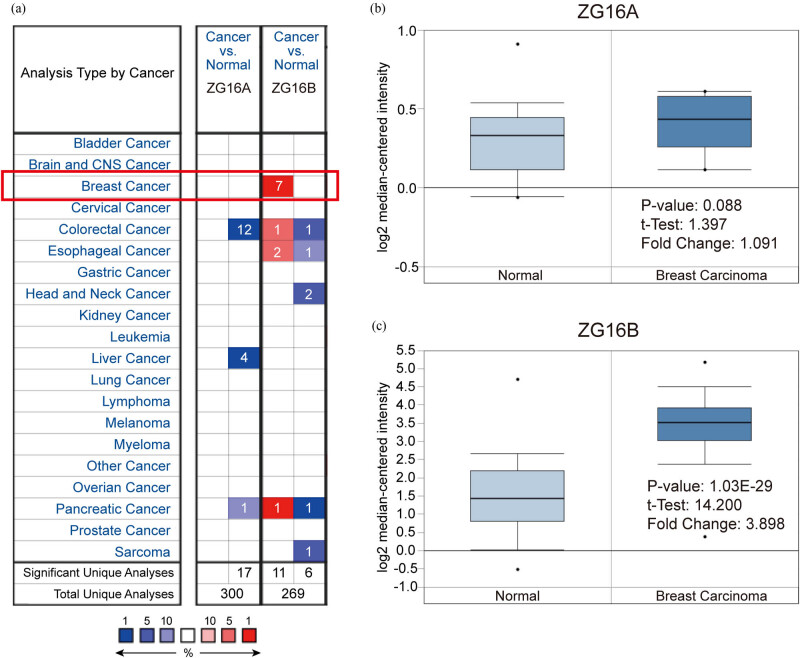
Analysis of ZG16A and ZG16B mRNA expression in different types of cancer (Oncomine). (a) The mRNA expression pattern of ZG16A and ZG16B in various cancers. The number in the grid represented the number of datasets that meet the fixed threshold (*p* < 1 × 10^−4^, fold change >2). The color of the grid represented if the gene expressed higher (red) or lower (blue) (cancer vs. normal). The color depth of the grid depended on the best gene rank percentiles in all genes detected in each analysis. Box plots derived from gene expression data in Oncomine comparing the mRNA expression of ZG16A (b) and ZG16B (c) in normal and breast cancer tissues.

Two kinds of zymogen granule proteins have been recognized in human body cells, including ZG16A and ZG16B. However, no report has announced their function and correlation in breast cancer yet. Oncomine database was used to analyze the different expression levels of ZG16A and ZG16B between multiple types of cancer and the corresponding normal tissues. Among all kinds of cancers, it was significant that the expression of ZG16B was upregulated in 7 analyses of 3 datasets from Curtis’ [47], Finak’s [48], and TCGA database which met the threshold. And yet no dataset showed high expression of ZG16A in breast cancer (Figure 1a). We analyzed the datasets uploaded by Ma [49] and Curtis [47]. No significant difference of ZG16A expression was observed between breast cancer tissue and normal tissue (*p* = 0.088) (Figure 1b), while the fold change of ZG16B expression was 3.898 (*p* = 1.03 × 10^−29^) (Figure 1c), which showed high significance.

**Figure 2:**
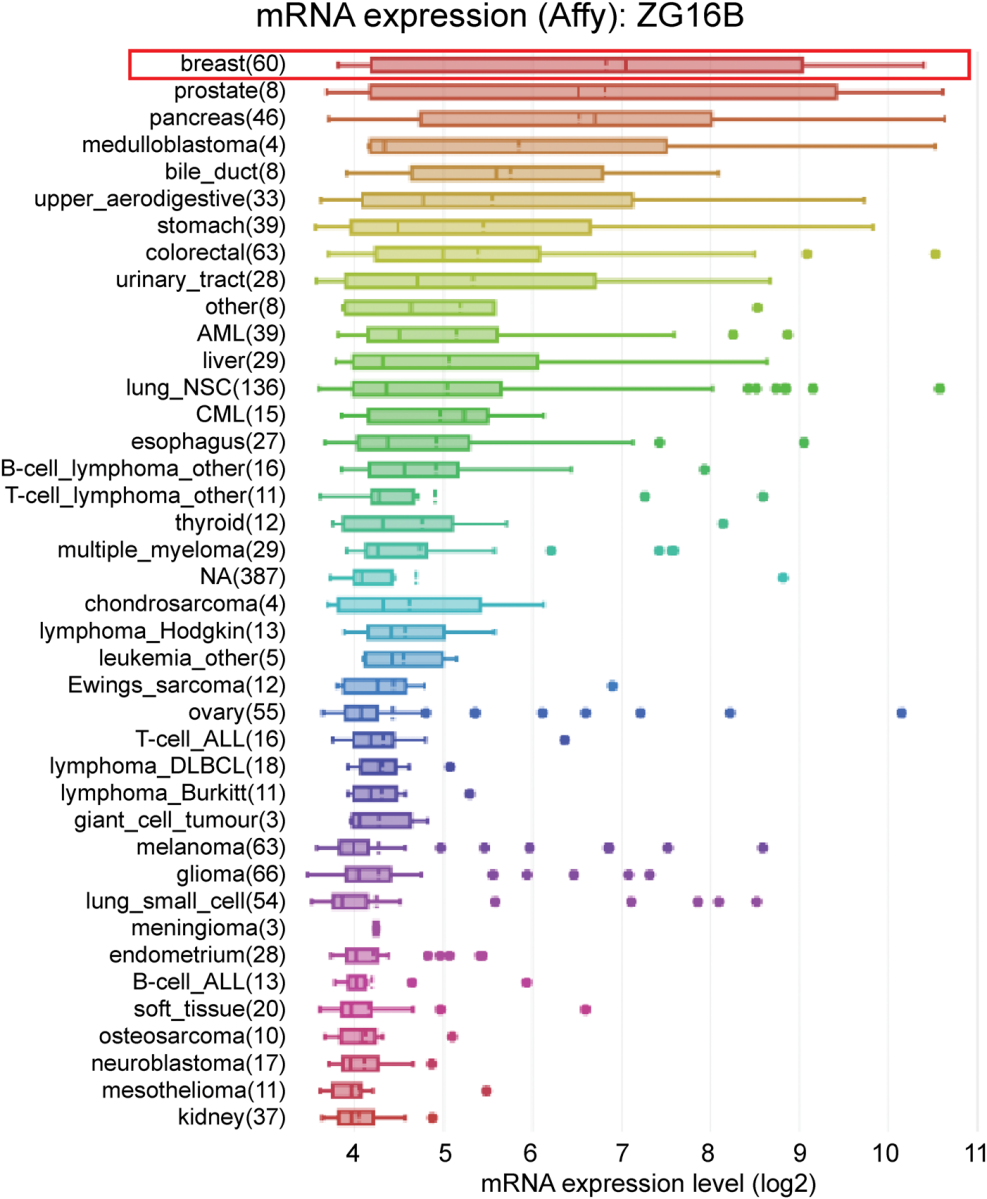
Analysis of ZG16B mRNA expression in cancer cells. ZG16B mRNA expression levels in 40 types of cancer cell lines were measured by Affymetrix gene chips and arranged from the highest to the lowest (CCLE analysis). ZG16B mRNA expression level was the first highest in breast cancer cells among all cancers.

Furthermore, in order to verify the high-level expression signal of ZG16B in breast cancer, we used CCLE analysis to detect the transcription level of ZG16B in multiple cancer cell lines. The results demonstrated that the transcription level of ZG16B was the highest among all types of cancer cell lines (Figure 2). These results indicated the special role of ZG16B in breast cancer.

**Figure 3:**
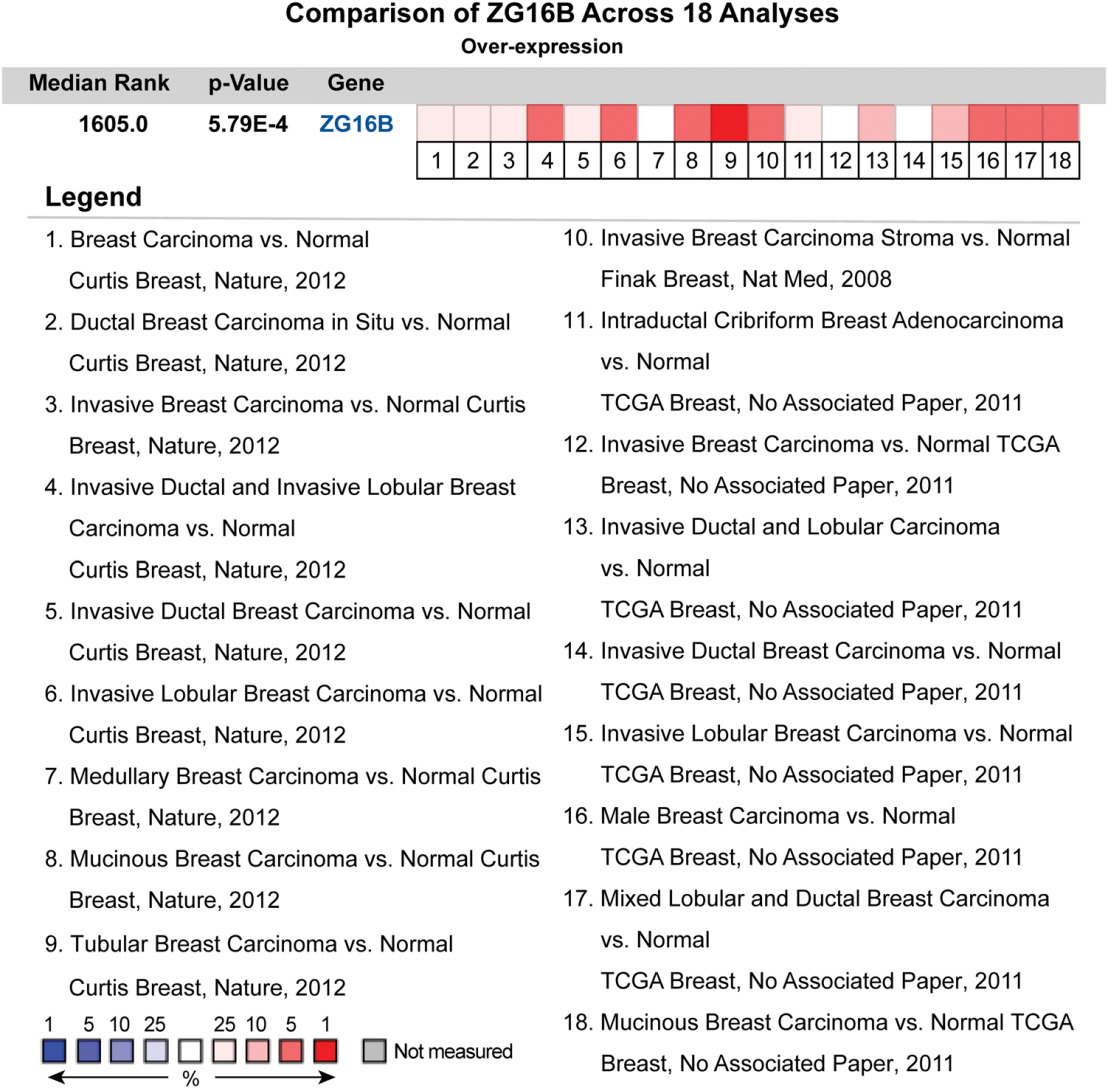
The pooled meta-analysis of gene expression profiling for ZG16B gene across 18 analysis. The color of the grid represented if the gene expressed higher (red) or lower (blue). The color depth of the grid depended on the median gene rank percentiles. The rank of ZG16B was the median rank across each of the analyses. The *p*-value of ZG16B was for the median-ranked analysis.

In consideration that the evidences described above indicate ZG16B expresses highly in breast cancer tissues and cell lines, we have done a further pooled meta-analysis including 2,780 samples from all the 18 researches of breast carcinoma in Curtis’ [47], Finak’s [48], and TCGA databases to confirm the high expression in general situation of breast cancer. The pooled meta-analysis confirmed that the mRNA upregulation of ZG16B was extremely significant in breast cancer (*p* = 5.97 × 10^−4^) (Figure 3). Persuasive testament was presented explaining the connection between the overexpression of ZG16B and breast cancer.

### 3.2 Analysis of ZG16B expression in different clinical features of breast cancer

**Figure 4:**
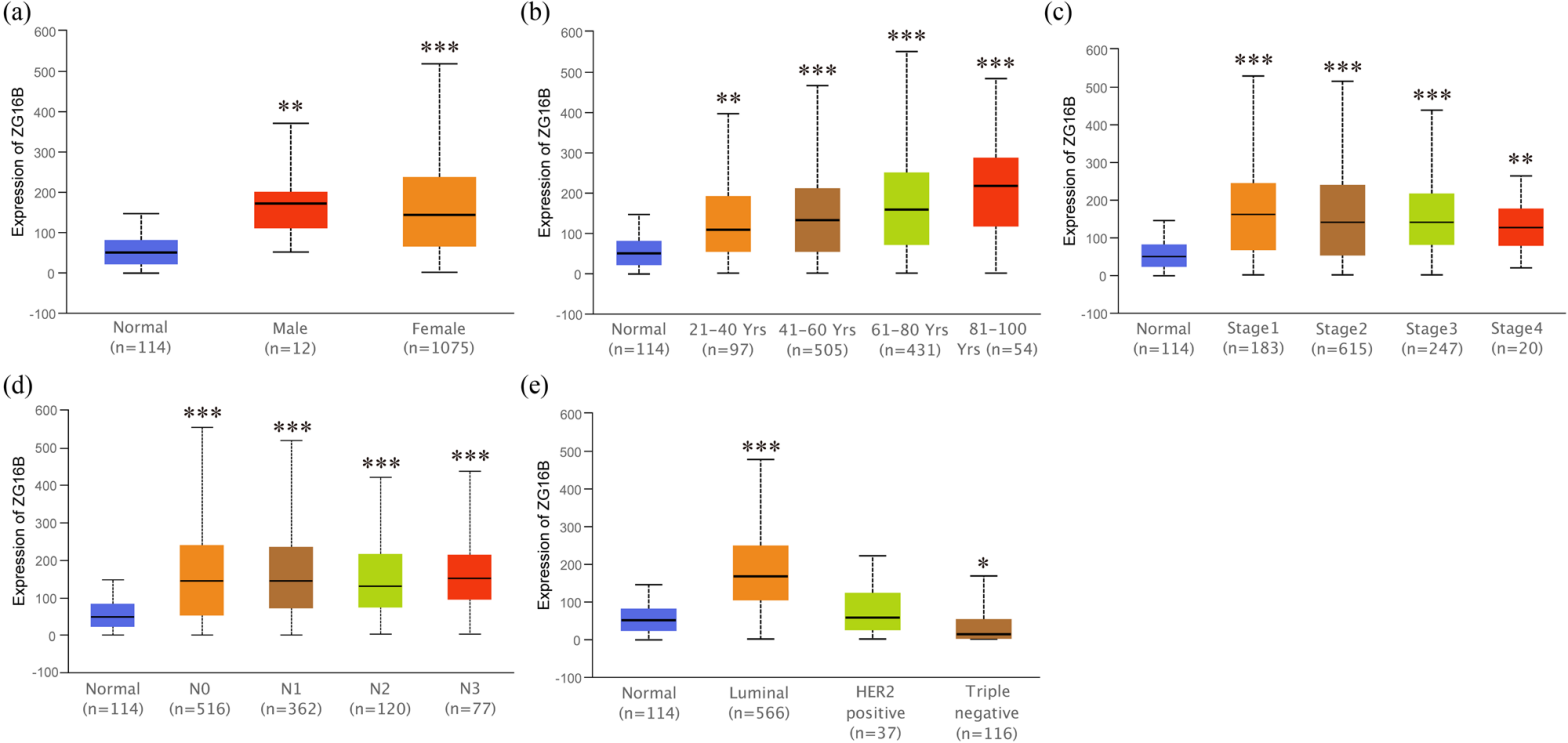
Analysis of ZG16B expression patterns in breast cancer patients with different clinical-pathologic features. Ualcan analysis showed the mRNA expression level of ZG16B in breast cancer patients with distinct gender (a), age (b), cancer stages (c), node metastasis status (d), and molecular subtypes (e). Asterisks were marked to show the significance of each breast cancer group compared with normal group (*p < 0.05, **p < 0.01, ***p < 0.001).

Since it showed that ZG16B indeed upregulated in breast cancer, in order to expound the expression pattern of ZG16B in breast cancer, Ualcan analysis was conducted to compare the expression level of ZG16B in different clinical indicators. The expression level of ZG16B increased in both female and rare male patients as shown in Figure 4a. Although ZG16B expression level is higher in all patient groups with different ages compared with normal groups, no significance could be found between groups (Figure 4b); and the situations could be classified in similar way by individual cancer stages and nodal metastasis status as shown in Figure 4c and d. Intriguingly, ZG16B expression level is significantly higher in luminal-like subtype (*p* = 1.624 × 10^−12^) and triple-negative subtype (*p* = 4.632 × 10^−2^) than in normal tissues, whereas no significance was shown in HER2-positive subtype (Figure 4e).

### 3.3 Hypomethylation of ZG16B promoter in breast cancer

**Figure 5:**
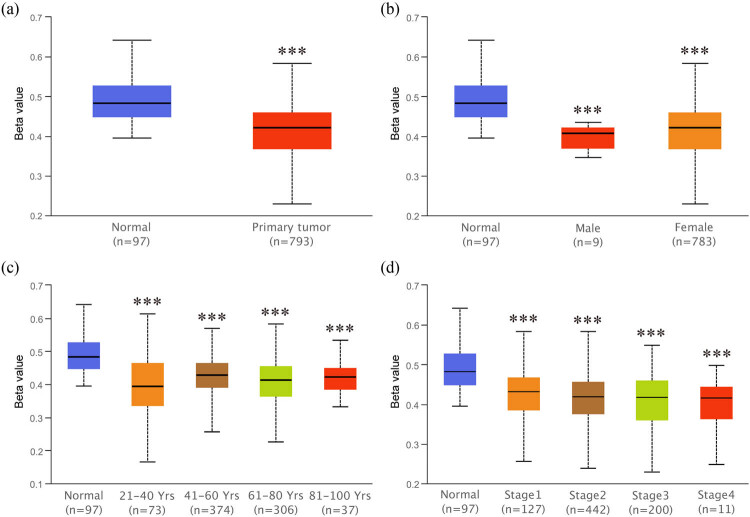
Analysis of ZG16B promoter methylation status in breast cancer patients with different clinical-pathologic features. Ualcan analysis showed the methylation level of ZG16B promoter in primary tumor of breast cancer (a) and in breast cancer patients with distinct gender (b), age (c), and cancer stages (d). Asterisks were marked to show the significance of each group compared with normal group (*p < 0.05, **p < 0.01, ***p < 0.001).

It has been reported that abnormal promoter hypomethylation induces irregular gene upregulation and then affects tumor progression [50,51,52]. Unsurprisingly, by the way of Ualcan, we found that the promoter methylation level of ZG16B significantly downregulated in primary breast tumor compared with that in normal tissues (Figure 5a). Patient groups with different gender, age, and nodal metastasis status showed similar results, when compared with the normal group; however, no significance was confirmed for within group comparison (Figure 5b–d). These results suggested that the overexpression regulatory mechanism of ZG16B in breast cancer might be the consequence of promoter demethylation.

### 3.4 Protein interactions and gene co-expression analysis

**Figure 6:**
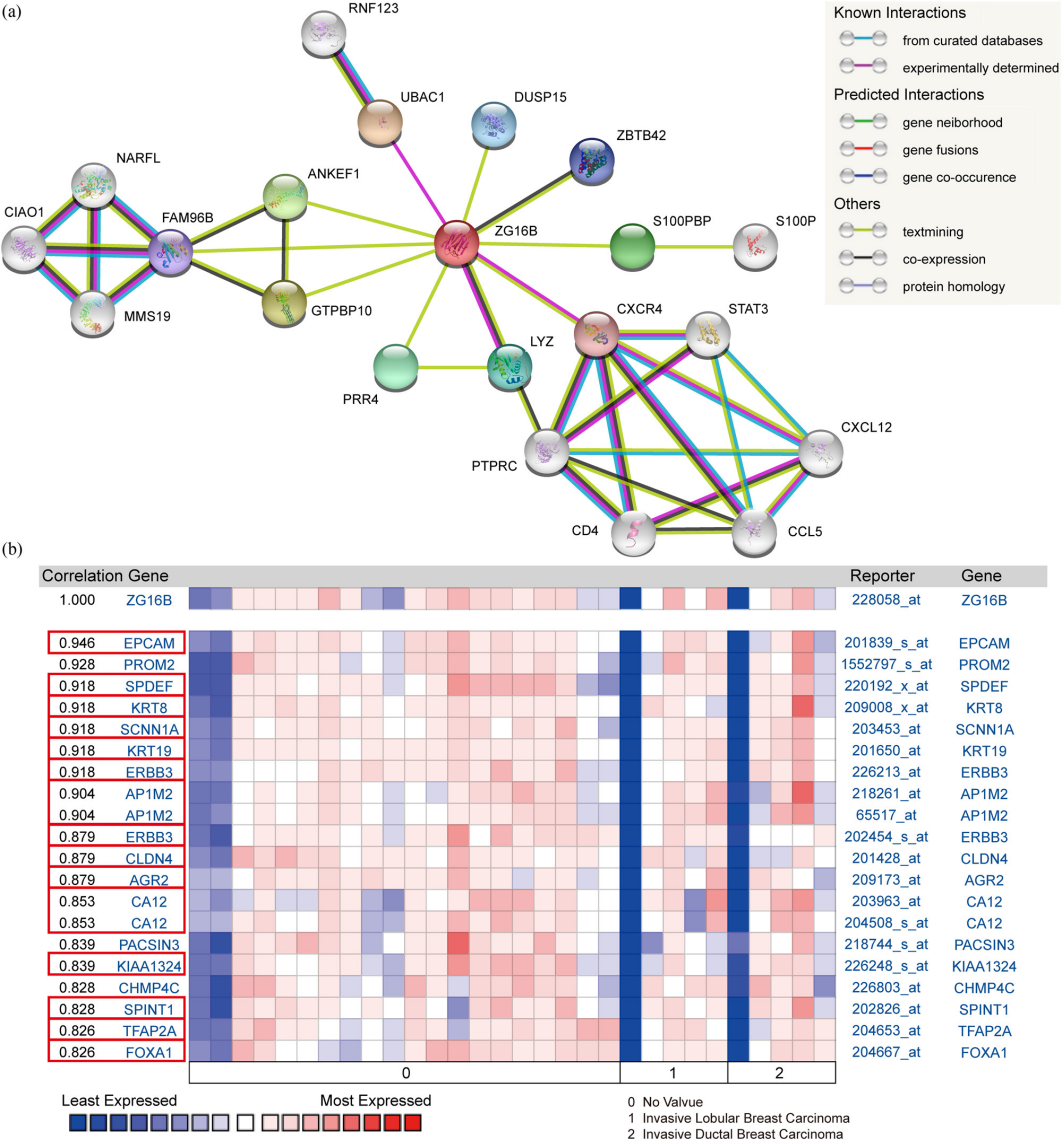
Protein interactions and gene co-expression analysis of ZG16B. (a) The protein interaction network of ZG16B was drawn by STRING analysis. The colored nodes in the network represented ZG16B and its first stage of interactors, and the white nodes represented the second stage of interactors. The physical or functional relations between these proteins are interpreted in the conventional signs. (b) The gene co-expression analysis of ZG16B in Turashvili’s work was conducted by Oncomine. The co-expression genes of ZG16B in breast cancer and their coefficients were marked by red rectangles.

STRING analysis was performed to extrapolate the possible mechanism of ZG16B action in breast cancer and find the interaction between ZG16B and other proteins. The protein network showed that UBAC1, LYZ, and CXCR4 are experimentally determined to have interactions with ZG16B, while ZBTB42 and LYZ co-expressed with it. In addition, STRING computationally predicted ZG16B might have physical or functional relation with DUSP15, ZBTB42, S100PBP, PRR4, GTPBP10, FAM96B, and ANKEF1 (Figure 6a).

In order to obtain more detailed information at genetic level, we have detected the co-expression genes of ZG16B in breast cancer through Oncomine database. As reported in Turashvili’s research [53], the expression of ZG16B is highly related to EPCAM (*r* = 0.946), SPDEF (*r* = 0.918), KRT8 (*r* = 0.918), SCNN1A (*r* = 0.918), KRT19 (*r* = 0.918), ERBB3 (*r* = 0.918), AP1M2 (*r* = 0.904), CLDN4 (*r* = 0.879), AGR2 (*r* = 0.879), CA12 (*r* = 0.853), KIAA1324 (*r* = 0.839), SPINT1 (*r* = 0.828), TFAP2A (*r* = 0.826), and FOXA1 (*r* = 0.826) as shown in Figure 6b. These demonstrate that ZG16B has strong interactions with various proteins involved in breast cancer formation, indicating the special role of ZG16B in breast cancer from another point of view.

### 3.5 Overexpressed ZG16B represents favorable prognosis of breast cancer patients

**Figure 7:**
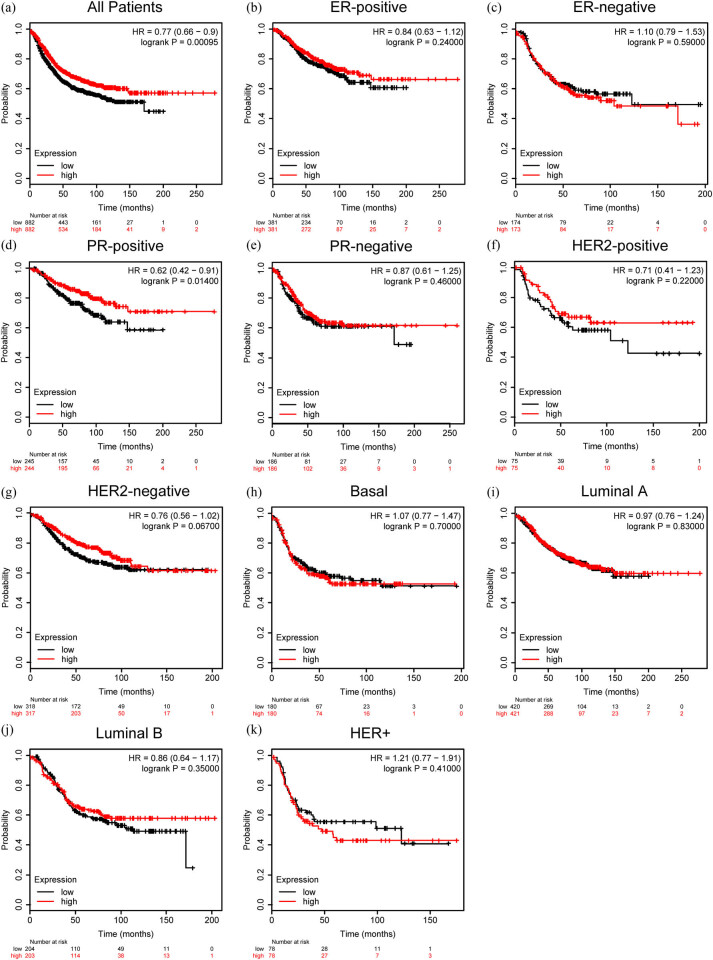
Prognostic value of ZG16B expression in different subtypes of breast cancer. The RFS curves were drawn by Kaplan–Meier plotter in all breast cancer patients (a) and different subtypes of breast cancer, ER-positive (b), ER-negative (c), PR-positive (d), PR-negative (e), HER2-positive (f), HER2-negative (g), Basal (h), Luminal A (i), Luminal (j), and HER2+ (k).

We further examined the impact of ZG16B on the prognosis of distinct molecular types of breast cancer by Kaplan–Meier plotter using the RFS as an indicator. Interestingly, higher expression of ZG16B represented a longer RFS for all breast cancer patients (HR = 0.77, *p* = 0.00095) (Figure 7a). The RFS of PR-positive patients also had a longer RFS as ZG16B expressed higher (HR = 0.62, *p* = 0.014) (Figure 7d), but there is no significance in other types of breast cancer (Figure 7b, c and e–k). These data indicated that ZG16B might be a general factor to mark a relatively favorable prognosis in breast cancer.

## 4 Discussion

Currently, breast cancer threatens the health of women around the world [3,54]. Various traditional early detection methods including X-ray, CT, MRI, and ultrasound have been used to diagnose breast cancer [55,56]. In recent years, biomarkers in breast cancer are found and used not only to predict the invasiveness [57], recurrence, distant metastases, and prognosis [58,59], but also to predict the response to chemotherapy [60], monitor the use of medicine [61], and being the target of therapy [62] to earn time and quality of life for patients. Fortunately, consensus has been reached by biomedical researchers to establish bioinformatics database such as Oncomine [63], TCGA database [64], CCLE database [65], and so on to discover and predict potential biomarkers and therapeutic targets which could be verified by experiments furthermore.

ZG16B as a biomarker of pancreatic cancer, ovarian cancer, etc. has no research correlated to breast cancer yet. In our work, we specially noticed in Oncomine database that ZG16B was also upregulated in several reports. In order to further verify the observation, CCLE analysis and pooled meta-analysis have been explored and confirmed that ZG16B indeed has high expression in tissues and cell lines of breast cancer, as shown in Figures 1–3. To figure out the expression pattern of ZG16B in breast cancer, Ualcan database analysis confirms that ZG16B upregulates in different clinical classifications, including gender, age, and nodal metastasis status; it also demonstrates that ZG16B has significantly high expression in luminal and triple-negative molecular subtypes of breast cancer, while this high expression is not found in HER2-positive subtype (Figure 4). In addition, the mechanism of demethylation epigenetic factor has been represented by Ualcan to explain the upregulation of ZG16B in breast cancer (Figure 5).

In order to explore the possible role of ZG16B in breast cancer, STRING analysis and Oncomine co-expression analysis have been performed successively and we found that ZG16B had close relationships with UBCA1, DUSP15, ZBTB42, S100PBP, PRR4, CXCR4, LYZ, GTPBP10, FAM96B, ANKEF1, EPCAM, SPDEF, KRT8, SCNN1A, KRT19, ERBB3, AP1M2, CLDN4, AGR2, CA12, KIAA1324, SPINT1, TFAP2A, and FOXA1 (Figure 6). Among these proteins related to ZG16B, LYZ [66] and PRR4 [67] have protective function in tears and various body fluids, which are corresponding to Perumal’s research [45,46]. S100PBP [68], PRR4 [69], ANKEF1 [39,70], EPCAM [71], SPDEF [72], KRT8, KRT19 [73], KIAA1324 [74,75], CXCR4 [76,77], AGR2 [78,79,80], SCNN1A [81], AP1M2 [82], CLDN4 [83], and ERBB3 [84,85] have been reported to have correlations with breast cancer or have been identified as biomarkers for diagnosis, metastasis, and prognosis of breast cancer and even as therapeutic targets. Interestingly, FAM96B is reported to inhibit VEGF receptor 2 promoter to restrain endothelium activity through the control of E2-2 expression [86]. SPINT1, which is one of the Kunitz-type serine protease inhibitors and also known as HAI-1, can inhibit hepatocyte growth factor function via regulation of HGFA, matriptase, and hepsin to inhibit the migration, proliferation, and invasion of breast cancer, indicating a good prognosis [87,88,89]. TFAP2A, also known as AP-2-α, is a transcription factor regulating the differentiation and proliferation of breast, the upregulation of which inhibits cell cycle, promotes apoptosis, and suppresses invasion in breast cancer via the regulation of various miRNAs [90,91,92]. FOXA1 high expression connects with good prognosis in breast cancer by relieving the epithelial-to-mesenchymal transition process and inhibiting migration, invasion, and metastasis [93,94]. These four genes co-expressed with ZG16B are corresponding to our exploration, the interaction of which may interpret that ZG16B high expression represents a favorable prognosis in breast cancer. In addition, DUSP15 is recognized as a special regulator gene for oligodendrocytes differentiation [95]. GTPBP10 is a mitochondrial protein as a ribosome biogenesis factor [96,97] correlated with multicentric glioblastoma [98]. The two genes co-expressed with ZG16B may suggest a potential function of ZG16B in nervous system.

Finally, to confirm whether ZG16B had clinical significance in breast cancer patients and investigate its effect on prognosis, we have done Kaplan–Meier plotter analysis to access the RFS survival curves in different molecular subtypes of breast cancer shown in Figure 7, and it has been observed that although most molecular subtypes except PR-positive subtype ZG16B seemingly don’t show apparent effect, ZG16B does represent a long RFS and good prognosis for all kinds of patients. Patients with PR-positive breast cancer had the most favorable prognosis among breast cancer subtypes with ZG16B high expression, suggesting its special role in PR-positive breast cancer. All the data discussed above confirm that ZG16B might be a potential biomarker of breast cancer which represents a favorable prognosis.

In conclusion, ZG16B upregulates in breast cancer and represents a favorable prognosis in patients. Furthermore, ZG16B has correlations with various biomarkers and factors of breast cancer, some of which have precisely inhibitory effect on breast cancer. More work and experiments are needed in order to further reveal more fundamental mechanism for its role in breast cancer.
